# Efficacy of Anterior Cervical Discectomy and Fusion Versus Cervical Disc Arthroplasty in the Treatment of Cervical Degenerative Disc Disease, Radiculopathy, and Myelopathy: A Systematic Review

**DOI:** 10.7759/cureus.74418

**Published:** 2024-11-25

**Authors:** Fakhri Awawdeh, Ali Salam, Varun Soti

**Affiliations:** 1 Neurological Surgery, Lake Erie College of Osteopathic Medicine, Elmira, USA; 2 Pharmacology and Therapeutics, Lake Erie College of Osteopathic Medicine, Elmira, USA

**Keywords:** anterior cervical discectomy and fusion (acdf), cervical degenerative disc disease, cervical disc arthroplasty, cervical stenosis, myelopathy, radiculopathy

## Abstract

Cervical degenerative disc disease (DDD) is a condition in which the discs in the neck deteriorate, causing symptoms including neck and arm pain, muscle weakness, and incoordination. In severe cases, it can lead to nerve and spinal cord compression, resulting in radiculopathy and myelopathy. This review aimed to assess the effectiveness of two surgical treatments, anterior cervical discectomy and fusion (ACDF) and cervical disc arthroplasty (CDA), for addressing cervical DDD, radiculopathy, and myelopathy. Following the Preferred Reporting Items for Systematic Reviews and Meta-Analyses (PRISMA) guidelines, a literature search was conducted in the PubMed and BioMed Central databases, from January to March 2024. Thirty-one studies were included, comparing the outcomes of ACDF and CDA in patients with cervical DDD, radiculopathy, and myelopathy. Data were analyzed to evaluate outcomes such as the Neck Disability Index (NDI), pain levels, neurological status, incidence of secondary surgeries, range of motion (ROM) maintenance, and occurrence of adjacent segment disease. CDA demonstrated comparable or superior clinical success to ACDF. Both the techniques showed similar improvements in NDI, pain levels, and neurological status during medium- and long-term follow-ups. CDA had lower rates of secondary surgeries and adverse events related to surgery or implants compared to ACDF. It also demonstrated a lower incidence of adjacent segment disease and better ROM preservation. The evidence supports CDA as a safe and effective alternative to ACDF for patients with cervical DDD, particularly those who may benefit from motion preservation. However, further long-term, multicenter randomized controlled trials are needed to provide more definitive guidance for clinical practice.

## Introduction and background

Cervical degenerative disc disease (DDD) refers to the deterioration of the intervertebral discs in the cervical spine. These discs, located between the vertebrae, allow for flexible movement and function as shock absorbers, mitigating spinal stress. Degeneration involves changes in disc characteristics, such as moisture loss, which diminishes elasticity and increases brittleness [1].

A primary factor in intervertebral disc degeneration is the loss of proteoglycans. These large molecules break down into smaller fragments, leading to a reduction in osmotic pressure within the disc matrix. This reduction causes a loss of water molecules and compromises the disc's mechanical properties. Discs with less water have a reduced capacity to withstand pressure, resulting in bulging and loss of height. The loss of proteoglycan also impairs the movement of other molecules into and out of the disc matrix. Serum proteins and cytokines infiltrate the matrix, affecting cells and accelerating degeneration. Alterations in disc shape can lead to collapse, decreased intervertebral space, and abnormal spinal movement [2].

Deteriorating discs may protrude into the spinal canal, causing radiculopathy (nerve root pressure) or myelopathy (spinal cord pressure), both of which can lead to painful and disabling symptoms. Nerve root compression may present as neck or arm pain, while spinal cord compression can cause neurological symptoms, such as weakness, paresthesia (numbness), tingling, and loss of balance and coordination [3].

Cervical DDD is a multifactorial condition that begins with disc deterioration and progresses to a cascade of adverse spinal events involving the facet joints and ligaments. Decreased disc height can increase the sagittal diameter, leading to various degrees of disc bulging or protrusion into the spinal canal. Osteophytes (bone spurs) may also creep into the canal, reducing space for the spinal cord and blood supply. The narrowing of the disc space increases stress on other spinal joints, such as the uncovertebral and facet joints, where osteophytes can further reduce space [3].

Cervical DDD with radiculopathy involves symptoms and signs related to the dysfunction of the cervical spinal nerves. An American population-based survey reported an average annual incidence rate of 83.2 per 100,000 (107.3 per 100,000 for males and 63.5 per 100,000 for females) for cervical DDD with radiculopathy [4].

Cervical DDD with myelopathy is less common than cervical nerve root compression. Anterior cervical discectomy and fusion (ACDF), first described by Smith and Robinson in 1958, has become the standard treatment for cervical disc herniations unresponsive to conservative therapy and cervical spondylotic radiculopathy or myelopathy. Despite advancements in anterior plate fixation and biologic substitutes, the procedure has remained largely unchanged due to its reproducible results and favorable long-term outcomes [5].

Over the past two decades, cervical disc arthroplasty (CDA) has emerged as a new approach in cervical spine surgery [6]. While ACDF has been the gold standard for 60 years, CDA challenges this paradigm. The discussion focuses on concerns regarding adjacent segment-level disease linked to ACDF and the limited range of motion (ROM) resulting from fusion. CDA, however, is a newer approach that offers significant motion preservation, especially in young patients, yet spine surgeons remain cautious [7].

This systematic review evaluates the effectiveness of ACDF and CDA in treating cervical DDD, radiculopathy, and myelopathy. It examines clinical evidence of symptom and pain improvement to determine the superior technique. The findings will provide valuable insights to aid spine surgeons in making informed decisions and developing personalized patient treatment plans.

## Review

Methods

**Figure 1:**
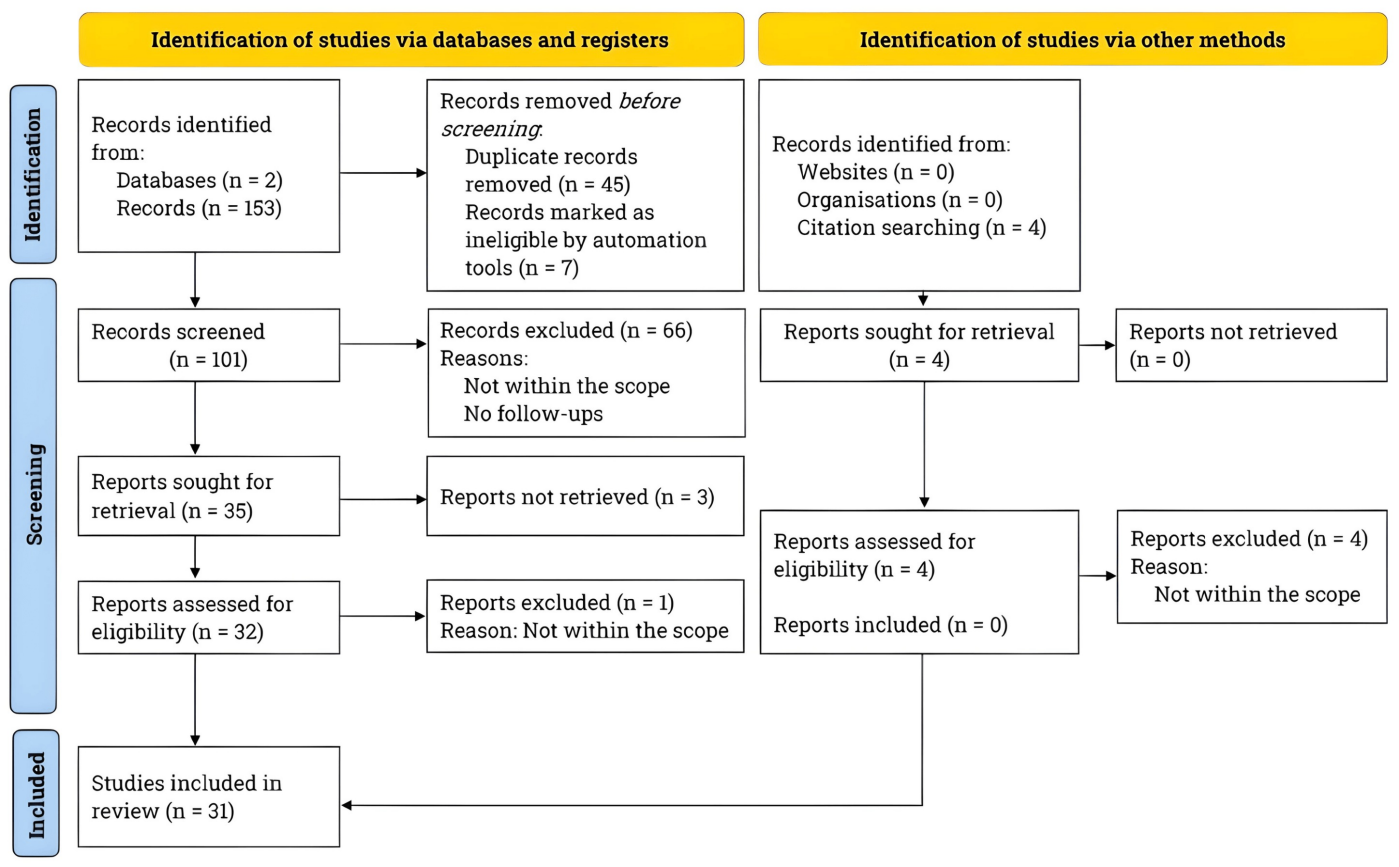
PRISMA flowchart for literature search and study selection. We adhered to the PRISMA guidelines and conducted an extensive literature review using the PubMed and BioMed Central databases. Our primary focus was on studies examining the postsurgical outcomes of cervical disc arthroplasty and anterior cervical discectomy and fusion in individuals with single-level and multilevel cervical degenerative disc disease, cervical radiculopathy, and cervical myelopathy. PRISMA: Preferred Reporting Items for Systematic Reviews and Meta-Analyses.

The literature search was conducted across two databases, PubMed and BioMed Central, from January to March 2024, following the Preferred Reporting Items for Systematic Reviews and Meta-Analyses (PRISMA) guidelines [8]. The search was not limited to studies published in the last 10 years to ensure a wide range of relevant literature. Figure 1 presents the PRISMA flowchart depicting the literature search process.

The keywords used were “cervical arthroplasty”, “cervical radiculopathy”, “myelopathy”, “fusion”, “spondylosis”, “anterior cervical discectomy and fusion”, “anterior cervical discectomy and fusion + degenerative disc disease”, and “cervical arthroplasty + degenerative disc disease”.

The literature search focused on identifying studies that analyzed the postoperative outcomes of patients with cervical DDD, radiculopathy, and myelopathy of the cervical spine with a preoperative indication for surgical intervention. The inclusion criteria were strictly limited to studies examining the success of ACDF and CDA and directly comparing postoperative outcomes of ACDF versus CDA. Table 1 presents the inclusion and exclusion criteria for the type of studies considered for this paper.

**Table 1:** Study selection criteria. The review encompassed pertinent research on the surgical outcomes of cervical disc arthroplasty and anterior cervical discectomy for treating patients with cervical degenerative disc disease (single- and multilevel), cervical radiculopathy, cervical myelopathy, herniated nucleus pulpous, and stenosis patients. The inclusion criteria were limited to studies published in English that met the study types outlined in the table.

Inclusion criteria	Exclusion criteria
Randomized controlled trials	Preclinical studies
Non-randomized controlled trials	Systematic reviews
Prospective clinical studies	Narrative reviews
Observational studies	Commentaries
Pilot studies	Opinions
Retrospective studies	
Meta-analyses	
Case series	
Case reports	

The process involved removing duplicate articles and screening titles and abstracts based on the inclusion criteria, followed by a full-text analysis to ensure all requirements were met. The initial search identified 38 studies, of which only 31 met all the inclusion criteria. These included seven studies that analyzed ACDF outcomes alone, seven that investigated CDA outcomes alone, and 17 that directly compared ACDF with CDA.

Surgical procedure overview of ACDF

The patient is positioned supine with the head in a neutral position and slightly hyperextended. Hyperextension is achieved by placing a roll transversely between the scapulae. The arms are folded against the body and retracted caudally to improve visualization of the cervical spine under lateral fluoroscopy. An incision of approximately 4.5 cm is made, starting at the midline or 1 cm lateral to it, extending to the medial edge of the sternocleidomastoid (SCM) muscle. The incision location may vary depending on the operative level and is guided by pre- and intraoperative fluoroscopy [9].

For an operation at the cervical (C) 4-5 level, the incision is made at the height of the upper edge of the thyroid cartilage. The skin and subcutaneous tissue are incised, exposing the platysma muscle. Blunt dissection is performed craniocaudally in the soft tissue to maintain the dissection plane and ensure optimal visualization. After incising the platysma, the SCM is identified laterally, while the midline muscles (sternohyoid, omohyoid (OH), and thyrohyoid) are identified medially. In the traditional approach to the subaxial cervical spine, blunt dissection is performed with Cloward retractors, medial to the SCM and neurovascular bundle (NVB) of the neck, which includes the internal carotid artery, internal jugular vein, and vagus nerve (cranial nerve X), along with its overlying pretracheal fascia [9].

The approach proceeds lateral to the trachea and esophagus. The OH muscle, which has two bellies and runs in a craniocaudal and mediolateral direction from the hyoid bone to the upper border of the scapula, must be identified. The OH muscle is then dissected and retracted superiorly for lower spinal segments (C5-C6 or C6-C7) or inferiorly for higher levels. Interfascial dissection and the use of silicone bands (cranial loop) facilitate OH eversion, maintaining retraction [9].

Important neurovascular structures crossing the surgical field must be avoided. At lower levels (C6-C7), the recurrent laryngeal nerve may be at risk. At upper levels (C3-C4, C4-C5, and C5-C6), the superior laryngeal nerve and the superior thyroid artery must be preserved due to their lateromedial and craniocaudal trajectories. The approach proceeds medial to the SCM and NVB, lateral to the trachea and esophagus, and cranial to the OH [9].

A surgical microscope is then used to optimize the visualization of the structures. The prevertebral plane is accessed by opening the pretracheal fascia medial to the NVB, avoiding injury to the trachea and esophagus. In the prevertebral plane, the prevertebral fascia is incised longitudinally. The longus colli muscle is identified and dissected subperiosteally and laterally to access the prevertebral space [9].

Once the midline is identified and the longus colli muscles are laterally retracted, autostatic retractors are placed. The anterior common vertebral ligament is identified and dissected, exposing the vertebral bodies and intervertebral discs. Intraoperative lateral fluoroscopy confirms the correct intervention level by positioning a dissector in the intervertebral disc space [9].

Anterior osteophytectomy and microdiscectomy are performed with a surgical microscope and high-speed drill. Microsurgical decompression of the spinal cord and corresponding cervical nerve roots is carried out. The discectomy is completed once the decompressed dura is visualized after performing a posterior osteophytectomy and opening of the posterior common vertebral ligament. High-speed drills and Kerrison rongeurs (sizes 2 and 3) are used for these maneuvers. Finally, the articular joints are drilled, and an appropriate interbody fusion device is introduced into the space [9].

Surgical procedure overview of CDA

The patient is positioned supine on a radiolucent table that allows for anteroposterior and lateral fluoroscopic imaging. The neck is kept in a neutral position, avoiding kyphosis and hyperlordosis. A transverse incision suitable for the Smith-Robinson approach (anterior approach) to the cervical spine is identified, typically along one of the several anterior skin folds for cosmetic reasons. Fluoroscopy is used to confirm the incision location. It is recommended to make the surgical approach contralateral to the side with the most notable symptoms (e.g., a right-sided approach for a patient with left-sided radiculopathy). The standard Smith-Robinson approach to the anterior cervical spine is used at the pathological level [10].

A 3- to 4-cm incision is made with good hemostasis. The platysma is split longitudinally and mobilized for later closure. The interval between the medial strap and the SCM muscles is exposed. The prevertebral space and fascia are dissected. The anterior longitudinal ligament and the target disc are identified and confirmed fluoroscopically. The midline of the vertebral bodies is marked with electrocautery above and below the target level. Modified Caspar-type pins are placed well above and below the target disc level in a parallel fashion, also parallel to the disc space, and confirmed fluoroscopically. An intradiscal measuring tool is used to ensure that the pins are placed at least 7 mm above and below the end plates of the target disc [10].

The initial discectomy is performed using a modified distraction technique, followed by index neurological decompression and bilateral neuroforaminal decompression. An incision is made in the anterior annulus, and the initial discectomy is carried out using microsurgical curettes and pituitary rongeurs to reach the posterior annulus and posterior longitudinal ligament. Intradiscal distraction is performed with the designated tool, and the distraction is maintained with the Caspar system. The primary distraction is not performed using the Caspar system; instead, the Caspar locking mechanism is used to retain the distraction achieved with the intradiscal distractor, preventing inadvertent loosening of the pins [10].

The posterior annulus and posterior longitudinal ligament are resected under magnification. Loupe magnification or an operating microscope is recommended. Complete neurological decompression is achieved by removing end-plate spurs, bilateral uncovertebral spurs, and herniated discogenic material. Bilateral decompression and complete removal of the posterior longitudinal ligament are recommended, even in patients with unilateral pathological involvement. End-plate flatness is achieved using a barrel-type burr [10].

Subsequently, the end plates and eventual arthroplasty device are sized, and the end plates are milled. The intradiscal decompression is visually assessed. The appropriate size of the arthroplasty device is determined using a tool designed to measure the anteroposterior length of the disc space. The sizing tool must fully cover the anteroposterior diameter of the index disc space to ensure the appropriate size of the arthroplasty device. Sizing is confirmed fluoroscopically, and additional use of the burr is considered for initial sizing. A side-cutting, barrel-type burr is preferred to evenly flatten the medial aspects of the uncovertebral joints. The biconvex electric milling tool is placed in the distracted disc space, and its placement is confirmed fluoroscopically. The ability to perform on-demand fluoroscopy with the radiology technician in the room is ensured [10].

The wound is irrigated using a small quantity of saline solution, and the milling tool is started. The distraction is slowly removed using the Caspar device. Compression is carefully applied to the end plates via the Caspar pins while the milling device remains continuously engaged. Once milling is completed, the device is stopped and removed. The wound is irrigated to remove bone and other debris, and hemostasis is achieved. Gelfoam patties or powder with thrombin are preferred, as bone wax could prevent the later incorporation of the arthroplasty device into the end plates. Excess hemostatic agents are removed. The end plates are inspected to confirm appropriate milling and verify the integrity of the neurological decompression. An appropriately sized arthroplasty device is prepared and opened. Under slight distraction, the device is carefully placed with the help of an insertion tool. Final radiographs are obtained before wound closure [10].

The wound and incision are closed in a manner typical for a Smith-Robinson approach. Final wound irrigation is performed, and the Caspar pins are removed. Bone wax is applied to the former Caspar pin sites, and hemostasis is confirmed. Final anteroposterior and lateral radiographs are obtained to confirm proper device placement. Typically, the device is correctly placed if one of the two anterior device flanges is positioned 1 mm anterior to the respective disc space. The platysma is approximated with a resorbable suture, the dermis is closed with a subcuticular resorbable suture, and a skin adhesive, such as 3M Steri-Strip (3M, St. Paul, MN, United States), is used for the final skin closure [10].

Clinical evidence for ACDF

In a prospective cohort study, Buttermann [11] assessed the long-term (more than 10 years) clinical outcomes of ACDF and compared them based on the primary diagnosis of disc herniation, stenosis, or advanced cervical DDD. The study included 159 consecutive patients who met the inclusion criteria and underwent a minimum 10-year follow-up. Of these, 90 patients underwent single-level ACDF and 69 underwent multilevel ACDF procedures. The primary diagnoses were herniated nucleus pulposus (HNP) (52 patients), stenosis (52 patients), and cervical DDD (55 patients). Over an 11-year follow-up period, 12 patients died, 11 were lost to follow-up, and three declined to participate in the study. All deceased patients passed away due to causes unrelated to their surgery or spinal conditions [11].

The study revealed that the HNP group had the shortest duration of symptoms (p < 0.05), the fewest levels treated (p < 0.05), and the lowest rate of concomitant low back pain (p < 0.05). The stenosis group was characterized by older age and the highest number of levels treated. The cervical DDD group demonstrated a higher proportion of females and the longest duration of symptoms (p < 0.05). Neurological improvement was observed across all groups (p < 0.05). Patients’ self-reported outcomes showed significant improvement across all relevant scales at follow-up compared to preoperative scores (p < 0.0001). Although the use of narcotics for pain management substantially decreased (p < 0.05), many patients reported continued long-term use of anti-inflammatory medications. Age, sex, and the number of levels treated did not significantly correlate with outcomes. Notably, the study found statistically significant improvement in all three diagnostic groups following ACDF (p < 0.05), with the HNP group experiencing relatively more remarkable improvement than the stenosis and DDD groups (p < 0.01) [11].

In a retrospective analysis, Burkhardt et al. [12] evaluated data from hospitalized patients who had undergone ACDF or ACDF with Caspar plating (CP) at least 17 years prior. The final evaluation included a standard questionnaire assessing their current neurological status, NDI, Odom’s criteria, modified EuroQol-5 dimensions, and their quality of life. A total of 122 patients were assessed and followed up after 25 years. Among these patients, 80 had undergone ACDF, while 42 had undergone ACDF with CP [12].

The researchers observed a significant decrease in the numeric pain rating scale for radicular pain following ACDF (p < 0.05) or ACDF with CP (p < 0.05) at follow-up compared to baseline. The clinical success rate for both procedures was 86.1% after a follow-up period of over 20 years. Postoperative findings revealed no significant differences between the two methods at follow-ups. Similarly, there were no notable disparities in the rates of repeat surgeries following ACDF or ACDF with CP due to cervical adjacent segment disease or DDD [12].

In a retrospective cohort study of an Iranian population, Haghnegahdar and Sedighi [13] evaluated 68 patients who had undergone ACDF for single-level cervical disc herniation from March 2006 to March 2011. The study’s outcome measures included a structured questionnaire addressing residual and new complaints, satisfaction with the operation, recent postoperative Visual Analog Scale (VAS), Japanese Orthopedic Association Myelopathy evaluation questionnaire, and follow-up cervical magnetic resonance imaging (MRI) [13].

The results indicated an 88.2% improvement in neck pain and an 89.7% improvement in arm pain. During the follow-up period of more than 12 months, no patients reported symptom recurrence. About 47.1% of the patients reported residual complaints, with sensory deficits being the most common. The study concluded that ACDF is an effective surgical technique for managing cervical disc herniation in the Iranian population [13].

In a prospective study, Gibson et al. [14] conducted research involving 53 patients who underwent multilevel ACDF. Of these patients, 28 received multilevel ACDF with cellular allograft, while 25 received ACDF with decellularized allograft. Patients in both groups substantially improved after undergoing procedures. There was significant improvement in clinical outcomes regarding NDI and VAS scores (for neck and arm pain) compared to baseline (p < 0.05). Nonetheless, the two groups had no statistically significant difference regarding these outcomes [14].

Among those who received cellular allograft, four patients experienced neck swelling, and three experienced transient dysphagia, which was resolved by the three-month follow-up. In the decellularized allograft cohort, one patient experienced an epidural hematoma, one required repeat surgery due to graft subsidence and foraminal stenosis, one experienced long-term miosis and mydriasis secondary to Horner’s syndrome, and the other required additional surgery for adjacent segment disease [14].

The study revealed a 92.8% fusion rate at the one-year follow-up for the 28 patients who received ACDF with cellular allograft, compared to an 84.0% fusion rate for the 25 patients who received ACDF with decellularized allograft. However, the difference in fusion rates between the two groups was not statistically significant. These findings suggest cellular allograft is a low-morbidity bone allograft option for multilevel ACDF, with minimal postoperative complications [14].

In a retrospective case-control study by Tasiou et al. [15], 114 patients who underwent anterior cervical procedures over a six-year period were analyzed. These patients had been diagnosed with cervical radiculopathy and/or myelopathy resulting from cervical DDD, cervical spondylosis, or traumatic cervical spine injury. The study revealed that 79% of the patients underwent ACDF, 12.3% underwent anterior cervical corpectomy and interbody fusion, 6.1% underwent ACDF with plating, 1.7% underwent odontoid screw fixation, and one patient underwent anterior removal of osteophytes for severe Forestier’s disease [15].

The mean follow-up time was 42.5 months. The success rate of all procedures was remarkable (p < 0.05). However, inter-group analysis showed that the success rate was not statistically significant. The overall complication rate was 13.2%. Specifically, adjacent intervertebral disc degeneration was observed in 2.7% of cases, while dysphagia occurred in 1.7%. Postoperative soft tissue swelling, hematoma, and dural penetration were observed in 1.7% of cases [15].

Moreover, esophageal perforation was noted in 0.9% of cases, along with aggravation of preexisting myelopathy, symptomatic recurrent laryngeal nerve palsy, mechanical failure, and superficial wound infection, each occurring in 0.9% of cases. It was emphasized that most complications resulting from anterior cervical spine surgery were minor and did not require further intervention. Despite the relatively low frequency of complications, these findings suggest the importance of considering potential complications when performing anterior cervical procedures such as ACDF [15].

In an observational study, Veeravagu et al. [16] investigated the long-term outcomes of single- versus multilevel ACDF procedures, focusing on revision rates and postoperative complications. The study analyzed data from 92,867 patients who underwent ACDF between 2006 and 2010. Among the 28,777 patients who were followed up for more than 24 months postoperatively, 12,744 underwent single-level ACDF, while 16,033 underwent multilevel ACDF [16].

The study results indicated that perioperative complications were significantly more common in multilevel procedures (p < 0.0001). However, patients who underwent single-level ACDF had higher rates of postoperative cervical epidural steroid injections (p = 0.01). The patients who underwent multilevel ACDF were 1.6 times more likely to have revision surgery within 30 days after the initial procedure (p = 0.02). Multivariate analysis at the two-year follow-up revealed that patients in the multilevel ACDF cohort were more likely to undergo surgical revision (p = 0.001), be re-admitted to the hospital for any cause (p = 0.007), and experience complications (p = 0.0003) [16].

The study concluded that increasing the number of levels fused during the initial surgery was associated with a higher rate of reoperations. Additionally, multilevel ACDF patients requiring further surgery often underwent more extensive revision procedures. Overall, this study found that cervical reoperations were more common in the multilevel ACDF cohort, with data indicating a higher incidence of complications and the need for revision surgeries involving more than two levels than those involving a single level [16].

Fountas et al. [17] conducted a retrospective study to examine the complications associated with ACDF. The study evaluated 1,015 patients who underwent first-time ACDF. These patients suffered from cervical spondylosis, cervical radiculopathy, and myelopathy related to cervical DDD. All patients were treated using the standard Smith-Robinson approach, employing either an autologous or allograft, with or without a plate. The mean follow-up time was 26.4 months [17].

The study reported a mortality rate of 0.1% (1 out of 1,015 patients) due to an esophageal perforation. The complication rate was 19.3% (196 of 1,015 patients). The most common complication observed was isolated postoperative dysphagia, occurring in 9.5% of the patients. The postoperative hematoma was noted in 5.6% of cases, requiring surgical intervention in only 2.4%. Symptomatic recurrent laryngeal nerve palsy was seen in 3.1% of cases. Other complications included dural penetration (0.5%), esophageal perforation (0.3%), worsening of preexisting myelopathy (0.2%), Horner’s syndrome (0.1%), instrumentation backout (0.1%), and superficial wound infection (0.1%). The study underscores the significant risk of postoperative complications associated with ACDF, necessitating timely management. These findings suggest exploring alternative surgical options, such as CDA, which may offer equally effective or potentially superior outcomes [17].

Table 2 presents the key characteristics of studies demonstrating the effectiveness of ACDF in treating cervical DDD, radiculopathy, myelopathy, HNP, and stenosis patients.

**Table 2:** Clinical evidence on the effectiveness of anterior cervical discectomy and fusion (ACDF) in treating cervical disc degenerative disease, radiculopathy, and myelopathy patients. The table highlights the key characteristics of studies demonstrating the effectiveness of ACDF in treating cervical disc degenerative disease, radiculopathy, myelopathy, herniated nucleus pulpous, and stenosis patients. The studies included were prospective, observational, and retrospective in design.

Author(s)	Study design	Sample size	Treatment level	Treatments	Follow-up (years)
Buttermann [11]	Prospective	159	One-level and multilevel	ACDF	11
Burkhardt et al. [12]	Retrospective	122	One-level	ACDF and ACDF with Caspar plating	17
Haghnegahdar and Sedighi [13]	Retrospective	68	One-level	ACDF	1
Gibsonet al. [14]	Prospective	53	Multilevel	ACDF with cellular allograft and ACDF with decellularized allograft	1
Tasiou et al. [15]	Retrospective	114	One-level	ACDF and ACDF with plating	3
Veeravagu et al. [16]	Observational	92,867	One-level and multilevel	ACDF	2
Fountaset al. [17]	Retrospective	1,015	One-level	ACDF, ACDF with allograft, and ACDF with plating	2

Clinical evidence for CDA

In a randomized controlled trial, Hou et al. [18] assessed the efficacy and safety of CDA using Mobi-C (ZimVie Inc., Westminster, CO, United States) for treating single-level cervical disc spondylosis. The study included 99 patients aged between 21 and 60 diagnosed with degenerative cervical disc spondylosis and single-level cervical DDD [18].

The study results revealed that CDA using Mobi-C and ACDF effectively treated patients and improved their clinical status during a five-year follow-up. However, intraoperatively, patients who underwent CDA experienced substantially lesser total blood loss than those who underwent ACDF (p < 0.001). A statistically significant disparity (p = 0.049) in the incidence of additional surgery was observed between the two groups, with one Mobi-C patient necessitating reoperation compared to seven ACDF patients requiring further surgery in the fourth year of the study follow-up [18].

Moreover, significant differences were found between the two groups in the ROM of the treated cervical segment, with patients in the ACDF group exhibiting a limited ROM compared to those in the Mobi-C group (p < 0.001) at the last follow-up. The study concluded that Mobi-C arthroplasty presents a safe and effective alternative to ACDF [18].

Radcliff et al. [19] conducted a prospective, multicenter, randomized clinical trial to assess the long-term outcomes of CDA using Mobi-C compared to ACDF in treating one and two-level cervical DDD. Patients diagnosed with symptomatic cervical DDD at C1 or C2 levels were randomized into two groups: one group underwent CDA using Mobi-C, and the other group underwent ACDF with allograft and anterior plate. The study followed 599 patients, of whom 164 were treated with one-level CDA, 225 with two-level CDA, 81 with one-level ACDF, and 105 with two-level ACDF. The study participants were followed up to 84 months postoperatively. The study outcome measures included overall success, NDI, VAS for neck and arm pain, segmental ROM, patient satisfaction, 12-item Short-Form Survey Mental Component Scores and Physical Component Scores (SF-12 MCS/PCS), complications, and subsequent surgery rates. The study reported follow-up rates ranging from 73.5% to 84.4% at seven years [19].

For two-level procedures, the overall success rates were higher in the CDA group (60.8%) compared to the ACDF group (34.2%) (p < 0.0001). For one-level procedures, the overall success rates were similar (CDA, 55.2%; ACDF, 50%). Patients in the CDA and ACDF groups significantly improved their NDI scores, VAS neck and arm pain scores, and SF-12 MCS/PCS scores compared to baseline scores (p < 0.0001) [19].

In the single-level cohort, more CDA patients reported being “very satisfied” (90.9%) than ACDF patients (77.8%) (p = 0.028). The adjacent secondary surgery rate was considerably lower in the single-level CDA group (3.7%) than in the ACDF group (13.6%) (p = 0.007). In the two-level CDA group, the NDI success rate was significantly greater in CDA patients (79.0%) than in ACDF patients (58.0%) (p = 0.001). The rate of subsequent surgery at the index level was lower in the CDA group (4.4%) than in the ACDF group (16.2%) (p = 0.001). Similarly, the CDA group had a lower rate of adjacent-level secondary surgery (4.4%) than the ACDF group (11.3%) (p = 0.03) [19].

The study findings demonstrated the clinical superiority of two-level CDA over ACDF and the comparable outcomes for single-level CDA and ACDF. Both procedures effectively reduced pain, with low rates of increased disability or neck pain. At the seven-year mark, the satisfaction rates for CDA and ACDF patients were impressive, with over 95% expressing high satisfaction with their treatment for cervical total disc replacement (TDR) and 88% for ACDF. The benefits of CDA over ACDF were more pronounced with two-level procedures, indicating a significant advantage for TDR in such cases [19].

Vaccaro et al. [20] conducted a multicenter prospective clinical trial to evaluate the long-term clinical effectiveness and superiority of CDA using Secure-C (Globus Medical, Audubon, PA, United States) compared to ACDF for one-level cervical DDD over a seven-year follow-up period. The trial enrolled 380 patients across 18 surgical sites, with 89 receiving CDA using Secure-C in a non-randomized group. Among the 291 randomized patients, 151 received Secure-C, and 140 underwent ACDF treatment. The overall success of the surgeries was assessed based on several criteria: at least a 25% improvement in NDI scores, no device failure necessitating revision, absence of significant complications, and, for ACDF patients, clinical imaging showing no pseudoarthrosis [20].

During the seven-year follow-up, four patients passed away, and 30 experienced adverse outcomes, including revisions, removals, additional fixation, and further operations at the initial level among the randomized groups. Pain assessments and NDI scores did not differ significantly between the CDA and ACDF groups. However, the overall success rate was considerably higher for the CDA group at 86.3% compared to 70% for the ACDF group (p <0.05). The study concluded that Secure-C was non-inferior to ACDF regarding long-term pain relief and demonstrated statistical superiority in overall success [20].

Pimenta et al. [21] conducted a prospective trial to evaluate the effectiveness of single-level versus multilevel CDA. The researchers recruited 229 patients with various cervical spine conditions, including HNP, spondylosis, and cervical radiculopathy or myelopathy. Patient progress was evaluated using self-assessment tools, the NDI, VAS scores, and Odom’s criteria. Following anterior cervical neurological decompression, 71 patients underwent CDA with a Porous-Coated Motion (PCM) Cervical Disc (NuVasive, San Diego, CA, United States) at a single level from C3-C4 to C7-thoracic (T) 1. Concurrently, 69 patients underwent 158 multilevel PCM cervical disc CDA surgeries, with 53 cases involving double-level surgeries, 12 cases involving three-level surgeries, and four cases involving four-level surgeries [21].

The data from the self-assessment outcomes revealed a notable improvement in multilevel cases. Specifically, there was a mean improvement of 52.6% in NDI for multilevel cases compared to a 37.6% improvement for single-level cases (p = 0.021). Similarly, the mean VAS improvement for multilevel cases (65.9%) surpassed that of single-level cases (58.4%), although this difference was not statistically significant. Odom scores also demonstrated progress in the multilevel group (93.9%) compared to the single-level group (90.5%) in the excellent, good, and fair categories [21].

Reoperation rates and the incidence of serious adverse events were comparable between single-level and multilevel CDA groups. Furthermore, the Kaplan-Meier implant survivorship analysis at the three-year mark for the cohort of 229 prostheses was 94.5% (confidence interval, 1.00-0.820). The study concluded that clinical outcomes for multilevel CDA were significantly improved compared to single-level CDA [21].

In a single-center study, Dejaegher et al. [22] evaluated the long-term impact of CDA using BRYAN Cervical Disc (Medtronic Sofamor Danek, Memphis, TN, United States). The study assessed 89 patients suffering from cervical radiculopathy and myelopathy who underwent CDA with the BRYAN Cervical Disc prosthesis. Clinical outcomes, including neurological success, NDI, neck pain, arm pain, SF-36 (PCS and MCS), and radiological follow-ups to assess the ROM, were determined periodically up to 10 years post-CDA. Adverse events and secondary surgeries were also recorded and evaluated [22].

The study followed 72 patients for 10 years. It was found that 89% of patients either maintained or improved their neurological state after the 10-year follow-up, with statistical significance (p < 0.05). The neurological success rate remained consistently above 80% at all time points, except at the four-year follow-up, when it decreased to 72%. At six, eight, and 10 years post-CDA, the neurological success rates were 83%, 84%, and 89%, respectively. Additionally, SF-36 PCS scores significantly improved at all follow-up points (p < 0.05). In contrast, SF-36 MCS scores only showed significant improvement at the four- and six-year follow-ups (p < 0.05), with no substantial change at the eight- and 10-year follow-ups. Significant improvements in NDI, neck pain scores, and arm pain scores were observed at all follow-up points (p < 0.05) [22].

At the 10-year follow-up, radiological assessments for ROM indicated that the average angular motion of the prosthesis was 8.6°. In 81% of patients, the prosthesis showed mobility, having more than 2° of angular motion. Throughout the study, 21 patients (24%) experienced new or recurring radiculopathy or myelopathy, most of which were managed conservatively. Eight additional spine surgeries were required for persistent or recurring symptoms, with two patients (2%) being reoperated at the index level and five patients (6%) at an adjacent level. The study demonstrated positive long-term clinical outcomes following the insertion of the BRYAN Cervical Disc prosthesis, with most prostheses maintaining mobility even after 10 years. This suggests that CDA effectively treats cervical radiculopathy or myelopathy [22].

Garrido et al. [23] conducted a randomized controlled trial to assess and compare the functional outcomes of patients who either received the BRYAN Cervical Disc prosthesis or underwent ACDF over a 48-month follow-up period. The study enrolled 47 patients in a multicenter, prospectively randomized trial. Functional outcomes were measured using the NDI, VAS scores for neck and arm pain, and SF-36 (PCS and MCS) scores. Complications and reoperations were also recorded [23].

Over the 48-month follow-up, the BRYAN arthroplasty and ACDF cohorts showed improved functional outcomes in NDI, VAS neck/arm pain scores, and SF-36 (PCS and MCS) scores. The mean preoperative NDI score for the BRYAN arthroplasty group was 51, which improved to 10 at 48 months postoperatively. In the ACDF group, the mean preoperative NDI score was also 51, improving to 16.7 at 48 months. The success rate for NDI improvement, defined as a 15-point or more significant increase, was 93.3% in the BRYAN group compared to 82.4% in the ACDF group [23].

Preoperative VAS neck pain scores were 76.2 for BRYAN arthroplasty and 80.6 for ACDF, which improved to 13.6 and 28.1, respectively, at 48 months. Arm pain scores also showed significant improvement from 78.8 to 10.8 in the BRYAN group and from 77.1 to 21.7 in the ACDF group, indicating superior postoperative pain reduction in the BRYAN arthroplasty group [23].

During the 48-month follow-up, the ACDF group experienced six secondary surgeries, including three (12%) for adjacent-level cervical DDD, one (4%) for remote-level DDD, and two for pseudarthrosis in the same patient. In contrast, the BRYAN group had only one secondary surgery (5%) for adjacent-level DDD. The study concluded that CDA with the BRYAN Cervical Disc was more favorable than ACDF, demonstrating a lower incidence of secondary surgeries and superior improvement in functional outcomes [23].

Jawahar et al. [24] conducted a prospective, randomized controlled clinical trial to compare the effectiveness of CDA (using Kineflex-C (SpinalMotion, Inc., Palo Alto, CA, United States); Mobi-C; and Advent Cervical Discs (Orthofix, San Antonio, TX, United States)) and ACDF in treating patients with one- and two-level cervical DDD. The study involved 93 patients with symptomatic cervical DDD who had not responded to conservative treatments. These patients were randomly assigned to receive either CDA (59 patients) or ACDF (34 patients), remaining unaware of their treatment group until after surgery. The researchers evaluated clinical success rates, symptom-free periods, and the incidence of adjacent segment disease [24].

Data collection included VAS scores, NDI, and cervical spine radiographs at various time points post-surgery. Surgical success was determined based on specific outcome measures, such as reductions in VAS and NDI scores, absence of neurological deficits, and no further intervention at the treated level. Adjacent segment disease incidence was assessed through radiological and neurophysiological examinations [24].

At a median follow-up of 37 months, 64 patients met the criteria for clinical success. The study found that NDI was a better predictor of outcome than the pain score. The incidence of adjacent segment degeneration was similar between the CDA and ACDF groups, with slightly higher rates among patients with concurrent lumbar DDD. The study also concluded that age, gender, smoking habits, and the number of levels treated did not have predictive values [24].

The findings suggest that CDA is comparable to ACDF in relieving symptoms for one- and two-level cervical DDD. Additionally, the risk of developing adjacent segment degeneration was similar for both procedures, though slightly higher in patients with concurrent lumbar spine DDD [24].

Table 3 presents the critical characteristics of significant studies showcasing CDA effectiveness in treating patients with cervical DDD, radiculopathy, myelopathy, HNP, and stenosis.

**Table 3:** Clinical evidence on the effectiveness of cervical disc arthroplasty (CDA) in treating cervical disc degenerative disease, radiculopathy, and myelopathy patients. The table displays the critical characteristics of studies showcasing the effectiveness of CDA in treating patients with cervical disc degenerative disease, radiculopathy, myelopathy, herniated nucleus pulpous, and cervical stenosis. These studies included randomized controlled trials (RCTs), prospective clinical studies (with a randomized arm), observational studies, and retrospective analyses.

Authors (year)	Study design	Sample size	Treatment level	Type of cervical disc	Follow-up (years)
Hou et al. [18]	RCT	99	One-level and multilevel	Mobi-C	5
Radcliff et al. [19]	RCT	599	One-level and two-level	Mobi-C	7
Vaccaro et al. [20]	Prospective	380	One-level	Secure-C	7
Pimenta et al. [21]	Prospective	229	One-level and multilevel	Porous-coated motion	3
Dejaegher et al. [22]	Prospective	89	One-level	BRYAN	10
Garrido et al. [23]	RCT	47	One-level	BRYAN	3
Jawahar et al. [24]	RCT	93	One-level and two-level	Kineflex-C, Mobi-C, and Advent	3

Clinical evidence directly comparing CDA versus ACDF

Hisey et al. [25] conducted a prospective randomized controlled trial to compare the efficacy and safety of one-level Mobi-C CDA with ACDF over a five-year follow-up period in patients diagnosed with cervical DDD with radiculopathy or myeloradiculopathy at one level from C3-C7 [25].

Two hundred forty-five patients were enrolled and randomized into two groups: 164 underwent CDA, and 81 underwent ACDF. The primary outcomes were assessed using overall success rates, which included the absence of reoperation, revision, or removal of the device, maintenance or improvement in neurological status, and no serious adverse events related to the device [25].

At the five-year follow-up, 61.9% of CDA patients and 52.2% of ACDF patients were classified as having overall success. While the differences were not statistically significant, the study demonstrated that CDA was non-inferior to ACDF in treating cervical DDD. The study outcomes indicated that CDA using the Mobi-C Cervical Disc is a viable alternative to ACDF, providing comparable clinical outcomes over five years [25].

Burkus et al. [26] conducted a multicenter, prospective, randomized controlled trial with a seven-year follow-up to evaluate the safety and effectiveness of CDA using the PRESTIGE Cervical Disc (Medtronic, Minneapolis, MN, United States) and compared it with ACDF. The study involved 541 patients diagnosed with single-level cervical DDD, randomly divided into two groups: 276 patients received CDA with the PRESTIGE Cervical Disc, and 265 received ACDF [26].

Clinical outcomes were assessed using NDI, SF-36 survey, neck and arm pain scores, and radiographic evaluations, which included a ROM and fusion assessment. Evaluations were conducted at specific intervals, including preoperative, intraoperative, and postoperative assessments at one and a half months, three months, six months, 12 months, 24 months, 36 months, 60 months, and 84 months [26].

Of the 541 patients, 395 (212 in the CDA group and 183 in the ACDF group) completed the seven-year follow-up. Both groups showed significant improvements in one and a half months, sustained over seven years. In the CDA group, mean NDI improvements from preoperative scores were 38.2 at 60 months and 37.5 at 84 months, while in the ACDF group, the corresponding means were 33.8 and 31.9. The differences between the CDA and ACDF groups at 60 and 84 months were significant (p = 0.014 and p = 0.002, respectively) [26].

The CDA group showed higher rates of maintained or improved neurological status, with 92.2% at 60 months and 88.2% at 84 months. This is remarkable compared to 85.7% and 79.7% in the ACDF group (p = 0.017 and p = 0.011, respectively). At 84 months, the percentage of patients who returned to work was similar in the CDA group (73.9%) and the ACDF group (73.1%). Postoperatively, the CDA implant maintained an average angular motion of 6.67° at 60 months and 6.75° at 84 months. The CDA group had a lower cumulative surgery rate at the index level (4.8% versus 13.7%, p < 0.001). These findings suggest that CDA using the PRESTIGE Cervical Disc has better clinical efficacy and safety outcomes over seven years than ACDF [26].

In a prospective randomized multicenter trial by Coric et al. [27], the clinical outcomes of CDA using the Kineflex-C Cervical Disc were compared with ACDF in patients with symptomatic single-level cervical DDD over a five-year follow-up period. The trial included 269 patients, 136 in the CDA group and 133 in the ACDF group. Validated outcome measures, including ROM, NDI, and VAS, were utilized, and patients were assessed preoperatively and at regular intervals postoperatively up to 60 months [27].

CDA and ACDF groups demonstrated significant improvements in mean NDI and VAS scores at six weeks post-surgery, which persisted throughout the 60-month follow-up compared to presurgical scores (p < 0.01). No significant differences in NDI and VAS scores were observed between the two groups over the 60-month follow-up period. The ROM in the CDA group significantly increased from preoperative values at the 12-month and 24-month follow-ups and remained significant through the 60-month follow-up (p < 0.01). The rates of reoperation/revision surgery and adverse events related to the device or surgery were not significantly different between the two groups [27].

Notably, at the 60-month follow-up, the composite clinical success rate was significantly higher in the CDA group compared to the ACDF group (77.2% versus 57.9%, p < 0.05). The trial concluded that while clinical outcomes for CDA and ACDF were comparable, CDA showed a significantly higher overall clinical success rate, suggesting its viability as an alternative to ACDF for appropriately selected patients with cervical radiculopathy [27].

In a study conducted by Donk et al. [28], the effectiveness of CDA for patients with symptomatic mono-level cervical DDD was compared to ACDF and ACD. This double-blind, randomized controlled trial, involving multiple centers, spanned a nine-year follow-up period. Evaluations were based on the NDI, McGill Pain Questionnaire Dutch Language Version (MPQ-DLV), SF-36 (PCS and MCS), and the reoperation rate, both pre-procedure and during the follow-up period. The MPQ-DLV also included the pain description and rating indexes. Patients described pain from a given list of adjectives, and the number of words chosen-total (NWC-T) was counted. The pain rating index-total (PRI-T) was calculated from the sum of the ranks corresponding to the chosen adjective [28].

A total of 142 patients were assigned to three groups: 50 in the CDA group, 47 in the ACDF group, and 45 in the ACD group. One patient in the ACDF group did not complete the final questionnaire, and another patient in the CDA group was switched to a cage implant during surgery due to the inability to place the disc prosthesis. According to the intention-to-treat principle, this patient was still included in the CDA group for analysis [28].

For the 140 patients, the median last follow-up period was 8.9 years, ranging from 5.3 to 12.2 years. The outcomes were influenced by the baseline score (p = 0.009), while gender, surgeon, time to enrollment, and age did not affect the results across the treatment groups. The primary outcome, NDI, showed a statistically significant improvement of 13.4 ± 0.8 points compared to the baseline at two years (p = 0.009). However, no statistically significant difference was observed between the three groups [28].

At the last follow-up, NDI had improved to 7.5 ± 8.5 from the baseline, with no statistically significant difference among the groups (p = 0.324). The only clear and statistically significant improvement in NDI was observed between the preoperative measurements and six weeks postoperatively (p < 0.05). There were no clinically relevant changes in NDI during the follow-up period. The mean difference in NDI between two years postoperatively and the last follow-up was 2.0 ± 0.7 (p = 0.009), with no statistically significant difference observed between the treatment groups [28].

At the last follow-up, 73.5% of patients treated with CDA, 60.9% with ACDF, and 57.8% with ACD had a good outcome, defined as an NDI score of ≤7, although this was not statistically significant (p = 0.239). Enrollment time, gender, age, and surgeon were unrelated to any clinically relevant outcome measurement [28].

Regarding the SF-36 summary scales, the mean PCS improvement at two years was 32.1 ± 2.5, with no statistical difference observed between treatment modalities at any follow-up (p = 0.873). The MCS improved by an average of 22.8 ± 2.1, again without a statistically significant difference between the groups at any follow-up (p = 0.874) [28].

VAS scores as part of the MPQ-DLV improved to 17.3 ± 24.0 overall, 9.2 ± 16.5 when complaints were minimal, and 24.4 ± 31.4 when maximal. Other measures, such as the NWC-T (4 ± 5) and PRI-T (6.3 ± 9.7), followed similar patterns, with none reaching statistical significance between treatment modalities (VAS at questionnaire completion, p = 0.429; VAS minimal pain, p=0.534; VAS maximal pain, p = 0.593; NWC-T, p = 0.690; PRI-T, p = 0.657). This randomized trial did not identify a significant difference between the three surgical modalities for treating single-level cervical DDD. However, considering the small sample size, these findings should be considered inconclusive [28].

Gornet et al. [29] conducted a prospective randomized controlled trial to evaluate the long-term (10-year) clinical safety and efficacy of CDA compared to anterior ACDF in treating cervical DDD at two adjacent levels. The trial enrolled 209 patients who received CDA and 188 patients who underwent ACDF using the low-profile titanium ceramic composite-based PRESTIGE-LP Cervical Disc [29].

At the 10-year follow-up, CDA’s overall success rate was 80.4%, compared to ACDF’s 62.2% (p < 0.05). CDA showed statistical superiority over ACDF for overall NDI and neurological success at 10 years, with a posterior probability of superiority (PPS) of over 93%. The improvements in NDI and neck pain scores were statistically superior for CDA compared to ACDF (PPS, 99% at two-year follow-up, 99.6% at five-year follow-up, and 99.5% at 10-year follow-up). Similarly, the neurological success rate in the CDA was superior to that of ACDF (PPS, 93.1% at two-year follow-up, 96.1% at five-year follow-up, and 95.6% at 10-year follow-up). Additionally, CDA demonstrated at least non-inferiority for all other study effectiveness measures, including disc height, and maintained mean angular ROM at treated levels for up to 10 years. The rates of severe heterotopic ossification (HO) did not significantly increase from seven to 10 years in the CDA group [29].

Furthermore, the CDA group had fewer serious adverse events related to implant-related or implant/surgical procedure-related and fewer secondary surgical procedures at both the index and adjacent levels than the ACDF group. The study demonstrated that the PRESTIGE-LP Cervical Disc implanted at two adjacent levels provided improved clinical outcomes and segmental motion 10 years after surgery and proved a safe and effective alternative to fusion [29].

In a study conducted by Janssen et al. [30], the efficacy and safety of CDA using ProDisc-C (Centinel Spine, West Chester, PA, United States) were compared against ACDF for treating single-level cervical DDD between the C3-C4 and C6-C7 levels. The study involved 209 patients from 13 sites randomly assigned to receive either ProDisc-C (103 patients) or ACDF (106 patients). The patients underwent preoperative evaluation, postoperative assessments at various intervals up to seven years, and subsequent annual evaluations [30].

The assessments encompassed the NDI, SF-36, postoperative neurological parameters, secondary surgical procedures, adverse events, neck and arm pain, and satisfaction scores. At the seven-year follow-up, 152 (92%) out of 165 patients underwent assessment, and no significant differences were found in demographic factors, follow-up rate, or patient-reported outcomes between the two groups [30].

The study findings revealed that both ProDisc-C and ACDF procedures effectively alleviated neck and arm pain, enhanced and sustained functionality, and improved health-related quality of life. Neurological status was either improved or maintained in 88% and 89% of patients in the ProDisc-C and ACDF groups, respectively. Notably, a smaller proportion of patients in the ProDisc-C group required secondary surgical procedures compared to the ACDF group (7% in the CDA group versus 18% in the ACDF group, p = 0.0099) after seven years. Furthermore, no substantial differences were observed in the rates of device-related adverse events between the two groups [30].

The study suggests that CDA with ProDisc-C is a safe and effective surgical option for addressing single-level symptomatic cervical DDD. The clinical outcomes following CDA with ProDisc-C were found to be comparable to those after ACDF. Additionally, patients treated with ProDisc-C demonstrated a lower likelihood of subsequent surgery, indicating that CDA may yield durable results and slow the progression of adjacent-level disease [30].

In a prospective randomized controlled trial by MacDowall et al. [31], the efficacy and long-term implications of CDA versus ACDF were investigated in patients undergoing surgery for cervical radiculopathy. Of the 153 patients, 83 underwent CDA, and 70 underwent ACDF. The study assessed outcomes after five years using patient-reported outcome measures, with the NDI score as the primary outcome. Motion preservation and HO were evaluated through radiography, while adjacent-segment pathology (ASP) was assessed using the MRI. The necessity for secondary surgical procedures was also considered [31].

The results showed a significant improvement in NDI scores within the CDA and ACDF groups compared to the baseline (p < 0.01). However, analysis between the two groups revealed no statistically significant difference at various follow-up points during the study. Notably, 54% of patients in the CDA group maintained motion at the operated cervical level, while 25% experienced spontaneous fusion. There was no notable distinction between the two groups regarding the proportion of patients needing additional surgeries (p = 0.11), as 21% of CDA and 10% of ACDF patients underwent secondary surgery. Five patients from each group underwent secondary surgery due to clinical ASP. The study showed that CDA and ACDF resulted in comparable clinical outcomes, a similarity that persisted throughout the five-year follow-up period [31].

Phillips et al. [32] conducted a prospective randomized controlled trial to evaluate the long-term outcomes of PCM Cervical Disc CDA compared to ACDF in patients with symptomatic single-level degenerative spondylosis between C3-C4 and C7-T1. The trial consisted of 293 patients randomly assigned to undergo either CDA (163 patients) or ACDF (130 patients). Patient-reported outcomes, including neck and arm pain measures, VAS scores, NDI, and general health and mental component scores, were assessed at the five-year mark [32].

The trial’s findings indicated that the CDA and ACDF groups significantly improved across all measured outcomes. Five years after the surgery, both groups showed considerable enhancements in patient-reported outcomes such as neck and arm pain VAS scores, NDI, and general health (36-SF PCS and MCS). The CDA group exhibited significantly higher mean scores for NDI (p = 0.001), neck pain (p = 0.002), general health (PCS, p=0.014; MCS, p=0.004), and patient satisfaction (p = 0.005) compared to the ACDF group [32].

Additionally, 2-7 years post-operation, CDA patients experienced a lower occurrence of device-related serious adverse events (0.5% for CDA versus 1.1% for ACDF) and secondary surgical procedures (3.3% for CDA versus 7.6% for ACDF). It was observed that adjacent-level degeneration was more frequent radiographically after ACDF (33.1% CDA versus 50.9% ACDF, p = 0.006). It was the primary reason for the increase in late-term secondary surgical procedures after ACDF. The study outcomes support PCM Cervical Disc CDA as an effective treatment for cervical DDD, providing comparable long-term clinical outcomes to ACDF [32]. 

Quinto et al. [33] conducted a meta-analysis comparing the 10-year outcomes of CDA and ACDF in patients with cervical DDD. The researchers included 12 randomized controlled trials involving 2,000 patients. The outcomes assessed encompassed reoperation rates, NDI, VAS scores, and the incidence of adjacent segment degeneration. The results indicated that CDA was associated with a lower incidence of adjacent segment degeneration and reoperation rates than ACDF. At the same time, both procedures demonstrated significant improvements in NDI and VAS scores [33].

The overall clinical outcomes between the two procedures did not show substantial differences. Consequently, the study concluded that CDA offers advantages over ACDF in lower adjacent segment degeneration and reoperation rates while providing comparable improvements in clinical outcomes [33].

The meta-analysis conducted by Peng et al. [34] evaluated the short and mid-to-long-term outcomes of CDA with ACDF in treating patients with cervical DDD. The study included 30 randomized controlled trials involving 8,784 patients. The findings revealed that the CDA group showed prolonged operative duration (p = 0.000) and better overall success, including short term, mid-term, and long term, with p = 0.000, respectively, indicating that the success rate of CDA was approximately double that of ACDF. Also, the CDA group exhibited neurological success (p < 0.05) and improvement in NDI (p = 0.000) in all follow-up periods [34].

The CDA group had a higher incidence of dysphagia and dysphonia during the short-term follow-up (p = 0.01). However, no difference was observed during mid-term and long-term follow-ups. The CDA group exhibited a lower incidence of adjacent segment disease during long-term follow-up and overall analysis (p = 0.008), with lower reoperation rates in all follow-up periods (p = 0.000). Hospital stay duration and blood loss were comparable in the CDA and ACDF groups. The incidence of implant events was also similar in all follow-up periods. The researchers found that CDA was more effective than ACDF in treating cervical DDD patients [34].

Wu et al. [35] conducted a meta-analysis that examined the efficacy of CDA in patients with multilevel cervical DDD and compared it with ACDF. Seven studies involving 702 patients with multilevel cervical DDD were included in the analysis. The findings revealed that patients undergoing CDA exhibited similar operative times, blood loss, NDI, and VAS scores compared to those undergoing ACDF. However, patients undergoing CDA demonstrated significant improvement in overall cervical spine motion at operated levels (p < 0.00001) and lower adjacent segment degeneration rates (p < 0.00001) [35].

Furthermore, the incidence of postoperative dysphagia was significantly lower in the CDA group compared to the ACDF group (p = 0.03). CDA was suggested to be a safe and effective surgical approach for treating multilevel cervical DDD. Nonetheless, the researchers emphasized the need for future long-term, multicenter, randomized, and controlled studies to further validate the safety and efficacy of multilevel CDA [35].

Findlay et al. [36] conducted a meta-analysis to compare the short-term, medium-term, and long-term outcomes after CDA and ACDF. The analysis included data from 14 randomized controlled trials with 3,160 patients predominantly with single-level cervical DDD and up to 10 years of follow-up [36].

The findings revealed that CDA demonstrated superiority over ACDF at the two-year mark and between four and seven years (p < 0.001). In the short term, CDA patients exhibited better patient-reported outcomes compared to ACDF (p < 0.001), with similar outcomes in the long term. Additionally, CDA showed better results in NDI, VAS scores, SF-36 PCS scores, dysphagia incidence, and patient satisfaction between four and seven years (p < 0.05) compared to ACDF [36].

Furthermore, the incidence of adjacent segment disease after CDA was lower at two years (short term) and between four and seven years (medium-to-long-term) than after ACDF (p < 0.05). The meta-analysis underscored the favorable outcomes of CDA over ACDF. The study suggests that continued long-term monitoring of patients who have undergone CDA may yield additional evidence supporting its widespread use in clinical practice for treating patients with cervical DDD [36].

Zheng et al. [37] conducted a comparative study to assess the effectiveness and safety of CDA versus ACDF in treating patients with single-level cervical disc herniation. The study included 145 patients, 64 undergoing CDA and 81 undergoing ACDF. Parameters such as surgery duration, intraoperative blood loss, VAS scores for arm and neck pain, ROM, Oswestry Disability Index (ODI), SF-36 (PCS and MCS) scores, and patient satisfaction were compared at baseline, pre-discharge, and at one, two, three, five, and eight years post-operation [37].

The findings indicated no significant differences between the two groups regarding surgery duration and intraoperative blood loss. Additionally, patient satisfaction rates did not differ significantly during the follow-up period. Moreover, there were no significant disparities between the groups in the incidence of temporary hoarseness (five patients in the CDA group versus seven in the ACDF group), dysphagia (eight patients in the CDA group versus 13 in the ACDF group) resolving within two weeks, and adjacent-level degeneration (two patients in the CDA group versus 10 in the ACDF group) post-surgery. Both groups experienced substantial postoperative benefits, as evidenced by considerable improvements in VAS scores (arm and neck pain), ODI, and SF-36 (PCS and MCS) scores before discharge compared to their baseline measurements (p < 0.05) [37].

In the intergroup analysis, significant differences emerged between the groups in VAS arm and neck pain scores, ROM, ODI, and SF-36 (PCS and MCS) scores after the surgery and during the eight-year follow-up. Patients in the CDA group demonstrated notably lower VAS scores, which gradually decreased significantly at various time points during the follow-up compared to those in the ACDF group (p < 0.01). All patients were immobilized before discharge. However, patients in the CDA group displayed significant ROM improvement faster than those in the ACDF group (p < 0.01) during the follow-ups [37].

ODI scores showed substantial decreases after the surgery in both groups, with a more marked improvement in the CDA group than in the ACDF group post-surgery and during the follow-up (p < 0.05). Patients in the CDA group exhibited significant improvements in SF-36 (PCS and MCS) scores compared to those in the ACDF group at various time points during the eight-year follow-up (p < 0.01). The study results indicate that replacing the entire disc may be more successful than ACDF for treating individuals with a single-level cervical disc protrusion [37].

Nunley et al. [38] conducted a prospective randomized controlled trial in which 155 patients were enrolled to compare 10-year outcomes between CDA (with the Mobi-C Cervical Disc) and ACDF for treating symptomatic cervical DDD. The trial was conducted at three centers [38].

At the 10-year follow-up, the study found that CDA demonstrated superiority over ACDF in several key outcome measures. The combined success rate was more remarkable in the CDA group at 62.4% compared to 22.2% in the ACDF group (p < 0.0001). Additionally, the cumulative risk of subsequent surgery at 10 years was significantly lower in the CDA group at 7.2% compared to 25.5% in the ACDF group (p = 0.001) [38].

Furthermore, the risk of adjacent-level surgery was markedly lower in the CDA group at 3.1% compared to 20.5% in the ACDF group (p = 0.0005), and the progression to radiographically significant adjacent segment degeneration at 10 years was also lower in the CDA group (12.9% in CDA versus 39.3% in ACDF, p = 0.006) [38].

Patient-reported outcomes at 10 years and changes from baseline favored the CDA group, with 98.7% of CDA patients reporting being “very satisfied” at 10 years than 88.9% of patients in the ACDF group (p = 0.05). The trial findings support the superiority of CDA over ACDF in treating symptomatic cervical DDD, as evidenced by superior clinical success, lower rates of subsequent surgery, and better neurologic outcomes. The results over 10 years indicate that CDA is a viable and successful surgical option for treating cervical DDD instead of fusion [38].

In a comprehensive analysis, Hu et al. [39] examined the clinical and radiological outcomes of single-level CDA and single-level ACDF in patients with preoperative reversible kyphosis due to cervical DDD. The study assessed patients who underwent single-level CDA and ACDF procedures between 2014 and 2018. The clinical outcomes were based on NDI, VAS scores, and Japanese Orthopedic Association (JOA) scores. Radiological outcomes were based on ROM, C2-7 Cobb angle, functional spinal unit (FSU) angle, and HO. These assessments were made pre-surgery and at subsequent follow-up intervals [39].

The study involved 80 patients, with 38 patients undergoing CDA (with an average follow-up duration of 39.8 months) and 42 undergoing ACDF (with an average follow-up duration of 37.6 months), all of whom presented with preoperative reversible kyphosis. Both groups showed significant improvements in JOA scores (p < 0.05), NDI (p < 0.05), and VAS scores (p < 0.05) compared to baseline. Upon intergroup analysis, there were no statistically significant variances concerning these clinical outcomes [39].

Post-surgery, CDA, and ACDF groups demonstrated significantly improved cervical alignment as indicated by the C2-7 and FSU angles, each with p < 0.05. Additionally, the C2-7 ROM was preserved in both groups (p < 0.05) compared to preoperative measurements, with no significant intergroup differences observed in these outcomes [39].

Moreover, the study findings indicated a mild decrease in segmental ROM in the CDA group, reducing from an average of 8.3° preoperatively to 5.1° during follow-up. In contrast, the ACDF group experienced a more pronounced decrease from an average of 8.1° preoperatively to nearly zero (p < 0.001). Furthermore, over 60% of patients in the CDA group developed HO, with nine cases classified as Grade Ⅲ and three cases as Grade Ⅳ, statistically significant compared to the ACDF group (p < 0.05) [39].

The study underscores that for patients with single-level cervical DDD and preoperative reversible kyphosis, both CDA and ACDF procedures may achieve comparable and effective clinical results. Moreover, CDA is non-inferior to ACDF regarding radiological outcomes related to cervical alignment despite the relatively higher incidence of HO formation, as observed in patients undergoing CDA [39].

In a retrospective study, Tracey et al. [40] evaluated and compared the clinical benefits, complications, and reoperation rates of CDA versus ACDF in patients with cervical DDD. The study included 259 patients, 171 undergoing CDA (average follow-up of 9.8 months) and 88 undergoing ACDF (average follow-up of 11.8 months) [40].

The study results indicated that 91.8% of CDA patients experienced complete relief of preoperative neurological symptoms. In the ACDF group, 86.4% had complete relief of all preoperative symptoms. Overall, most patients in both groups could return to their preoperative activity level, with 92.4% of CDA patients and 84.0% of ACDF patients from the active-duty military population returning to active duty after surgery [40].

Postoperatively, persistent posterior neck pain was observed in 15.4% of the CDA group and 12.5% of the ACDF group. The complication rate was 9.9% in the CDA group, including recurrent laryngeal nerve injury (2.9%), persistent dysphagia (5.8%), superficial infection (0.6%), and nerve root injury (0.6%). For the ACDF group, the complication rate was 9.1%, with occurrences such as persistent dysphagia (3.4%), pseudoarthrosis (3.4%), spinal cord injury (1.1%), and dural tears (1.1%) [40].

The reoperation rate was 3.5% in the CDA group, encompassing procedures such as posterior decompression and fusion, adjacent segment degeneration requiring surgery, and conversion of CDA to ACDF. In the ACDF group, the reoperation rate was 5.7%, involving procedures such as posterior decompression fusion and revision ACDF [40].

Based on the study findings, both CDA and ACDF demonstrate favorable and comparable clinical outcomes for patients with cervical DDD. The high percentage of patients experiencing complete relief of preoperative symptoms and the ability of most patients to return to their preoperative activity level in both groups highlight the overall effectiveness of both procedures. However, it is critical to note that both methods were associated with postoperative complications at similar rates and carried a risk of reoperation [40].

Lavelle et al. [41] conducted a multicenter, prospective randomized trial to assess the 10-year safety and efficacy of CDA using the BRYAN Cervical Disc. The trial encompassed 232 patients with symptomatic cervical DDD, among whom 128 underwent CDA and 104 underwent ACDF [41].

Upon the 10-year follow-up, the CDA group exhibited a considerably higher overall success rate (81.3%) than the ACDF group (66.3%), with a statistically significant p-value of 0.005. The incidence of subsequent surgeries at adjacent levels was lower in the CDA group (9.7%) than in the ACDF group (15.8%), albeit not reaching statistical significance [41].

The patients in the CDA group demonstrated a significant improvement in NDI compared to the ACDF group (p = 0.010). Although the CDA and ACDF groups experienced improvements in VAS scores (neck and arm pain), the difference was not statistically significant. Furthermore, the SF-36 scores, particularly PCS, exhibited marked enhancement in the CDA group compared to the ACDF group (p = 0.018) [41].

Serious adverse events were comparable and reported in 4.1% of patients in the CDA groups and 4.9% in the ACDF group. However, the average angular motions at the index level for the BRYAN Cervical disc in the CDA group were notably higher at 8.69° compared to 0.60° in the ACDF group [41].

The study results highlight that CDA provides long-term preservation and maintenance of motion compared to ACDF. Although there was a tendency toward fewer adjacent segment surgeries with the BRYAN Cervical disc in the CDA group, it did not achieve statistical significance. The substantial improvement in CDA NDI scores suggests potential long-term success for CDA compared to ACDF [41].

Table 4 summarizes the key characteristics of studies and findings comparing the safety and effectiveness of ACDF versus CDA in treating cervical DDD, radiculopathy, and myelopathy patients.

**Table 4:** Safety and effectiveness of anterior cervical discectomy and fusion (ACDF) versus cervical disc arthroplasty (CDA) in treating cervical disc degenerative disease, radiculopathy, and myelopathy patients. The studies that directly compared ACDF with CDA included randomized controlled trials (RCTs), prospective studies, observational studies, retrospective studies, comparative studies, and meta-analyses.

Authors (year)	Study design	Sample size	Treatment level	Treatments	Follow-up (years)	Was CDA safer and more effective than ACDF?
Hisey et al. [25]	RCT	245	One-level	CDA (Mobi-C) vs. ACDF	5	Outcomes were comparable
Burkus et al. [26]	RCT	541	One-level	CDA (PRESTIGE Cervical Disc) vs. ACDF	7	Yes
Coric et al. [27]	RCT	269	One-level	CDA (Kineflex-C) vs. ACDF	5	Yes
Donk et al. [28]	RCT	142	One-level	CDA (BRYAN Cervical Disc) vs. ACDF	9	Outcomes were comparable
Gornet et al. [29]	RCT	209	One-level	CDA (PRESTIGE-LP Cervical Disc) vs. ACDF	10	Yes
Janssen et al. [30]	RCT	209	One-level	CDA (ProDisc-C Cervical Disc) vs. ACDF	7	Outcomes were comparable
MacDowall et al. [31]	RCT	153	One-level	CDA (Discover Cervical Disc) vs. ACDF	5	Outcomes were comparable
Phillips et al. [32]	RCT	293	One-level	CDA (Porous-Coated Motion) vs. ACDF	5	Yes
Quinto et al. [33]	Meta-analysis	2,000	One-level	CDA vs. ACDF	10	Outcomes were comparable
Peng et al. [34]	Meta-analysis	8,784	One- and two-level	CDA (Advent, BRYAN, Discover, Kineflex-C, Mobi-C, PRESTIGE, PRESTIGE-LP, Porous-Coated Motion, and ProDisc-C Cervical Disc) vs. ACDF	10	Yes
Wu et al. [35]	Meta-analysis	702	Multilevel	CDA vs. ACDF	>1.5	Yes
Findlay et al. [36]	Meta-analysis	3,160	One-level and multilevel	CDA vs. ACDF	10	Yes
Zheng et al. [37]	Comparative	145	One-level	CDA (BRYAN Cervical Disc) vs. ACDF	8	Yes
Nunley et al. [38]	RCT	155	One- and two-level	CDA (Mobi-C Cervical Disc) vs. ACDF	10	Yes
Hu et al. [39]	Comparative	80	One-level	CDA vs. ACDF	>3	Outcomes were comparable
Tracey et al. [40]	Retrospective	259	One-level	CDA vs. ACDF	>1	Yes
Lavelle et al. [41]	RCT	232	One-level	CDA (BRYAN Cervical Disc) vs. ACDF	10	Yes

Discussion

This comprehensive systematic review conducted an in-depth analysis to evaluate the clinical outcomes of CDA compared to ACDF across diverse studies with varying follow-up durations and methodologies. The gathered data offer a comprehensive understanding of surgical methods’ efficacy, safety, and long-term ramifications in treating cervical DDD.

The reviewed studies consistently indicate that CDA is no less effective than ACDF in overall clinical success. For example, Hisey et al. revealed comparable success rates between CDA and ACDF at the five-year mark, implying that CDA presents a viable alternative for single-level cervical DDD [25]. Similarly, Burkus et al. demonstrated that CDA with the PRESTIGE Cervical Disc exhibited superior improvements in NDI and neurological status outcomes over seven years compared to ACDF [26]. These findings are further supported by Coric et al. and Gornet et al., who observed higher clinical success rates in the CDA groups at five- and 10-year follow-ups, respectively [27,29].

The available clinical evidence suggests that CDA is linked with a lower incidence of secondary surgeries and adverse events associated with the surgical procedure or implants. For example, Janssen et al. found that patients undergoing CDA with the ProDisc-C had a lower occurrence of secondary surgical procedures than those who underwent ACDF [30]. This trend is further substantiated by Nunley et al., who reported significantly lower cumulative risks of subsequent surgeries in the CDA group over 10 years [38].

Preserving the ROM at the operated levels is a crucial advantage of CDA, as emphasized across multiple studies. This is essential in safeguarding overall cervical spine flexibility and potentially mitigating the risk of adjacent segment disease. MacDowall et al. observed that a significant proportion of patients in the CDA group maintained motion at the operated level, which is associated with a lower rate of adjacent segment disease than ACDF [31]. Additionally, the meta-analyses conducted by Peng et al. and Wu et al. corroborated that CDA patients experienced lower incidences of adjacent segment disease and higher overall cervical spine motion postoperatively [34,35].

Patient-reported outcomes, such as satisfaction and quality of life measures, favored CDA. Phillips et al. found that CDA patients reported higher satisfaction and better scores in neck disability and general health measures than those who underwent ACDF [32]. This affirmative feedback aligns with the long-term satisfaction rates reported by Nunley et al., wherein a majority of CDA patients expressed being “very satisfied” with their outcomes after 10 years [38].

While the findings are promising, several studies noted limitations such as small sample sizes, potential biases in patient selection, and variations in surgical techniques and prosthetic devices used. For instance, Donk et al. highlighted the inconclusiveness of their results due to the small sample size and lack of statistically significant differences between the treatment groups [28]. Future research endeavors should concentrate on large-scale, multicenter trials to offer more robust evidence and address these limitations.

Limitations

This systematic review encountered potential limitations stemming from the scarcity of randomized controlled clinical trials directly comparing CDA to ACDF. The studies on CDA were affected by various constraints, including small sample sizes, particularly in the CDA arm of the clinical trials, and a lack of reporting on subsequent surgical interventions to address post-CDA complications. Moreover, many studies had relatively short follow-up periods, curtailing the assessment of CDA’s efficacy and success over long-term follow-ups, impeding capturing potential changes in failure rates and patient outcomes. Besides, a limited number of prospective investigations of CDA’s application in multilevel cervical DDD cases were available.

## Conclusions

The evidence strongly supports CDA as a safe and effective alternative to ACDF for treating cervical DDD. CDA shows comparable, if not superior, clinical outcomes in terms of overall success rates, neurological improvements, and patient satisfaction over both medium- and long-term follow-ups. Additionally, CDA is associated with fewer secondary surgeries, better maintenance of cervical spine motion, and a lower incidence of adjacent segment disease than ACDF. Considering these compelling findings, CDA should be viewed as a viable surgical option for appropriately selected patients with cervical DDD, particularly those who may benefit from motion preservation. However, further long-term, multicenter randomized controlled trials are required to provide more definitive guidance for clinical practice and substantiate these results.
